# Explore and Analyze the Composition and Characteristics of Intestinal Microbiota between Gastric Cancer Patients and Healthy People

**DOI:** 10.1155/2022/5834293

**Published:** 2022-09-08

**Authors:** Fang He, Fang Wang, Jiali Yang, Shaoqi Yang

**Affiliations:** Department of Gastroenterology, General Hospital of Ningxia Medical University, Yinchuan, Ningxia, China

## Abstract

Gastric cancer is one of the most common malignant tumors in the world. As the intestine is downstream of the digestive tract, the occurrence of gastric cancer may have a certain significant impact on it. Therefore, it is particularly important to find out the intestinal bacteria closely related to gastric cancer, to identify the specific flora related to gastric cancer, and to maintain the stability of the core structure of intestinal microecology in patients with gastric cancer. Based on this, the fecal samples of gastric cancer patients and healthy people were collected, and the diversity and composition of intestinal flora in patients with gastric cancer were analyzed by 16S rRNA sequencing technology. We found that there was no significant difference in the diversity and abundance of intestinal flora between gastric cancer patients and healthy people. The relative abundance of *Faecalibacterium*, *Bifidobacterium,* and *Subdoligranulum* in the intestinal tract of patients with gastric cancer was significantly lower than that in healthy people, while the relative abundance of *Enterococcus*, *Streptococcus,* and *Bacteroides* was increased. This study found that there were six kinds of intestinal microflora closely related to the occurrence of gastric cancer, which provided a theoretical basis for further exploring the pathogenesis of gastric cancer.

## 1. Introduction

Gastric cancer (GC), as one of the most common malignant tumors in the world, has about 1 million new cases and more than 700,000 deaths due to GC every year, which seriously threatens the life and health of the majority of people [1–3]. In China, the mortality rate of GC ranks second among all malignant tumors. At present, radical surgery is still the main treatment for GC [4]. After decades of research on the pathogenesis and progression mechanism of GC, dietary factors, environmental factors, *Helicobacter pylori* (*H. pylori*) infection, and genetic factors are considered potential risk factors [5, 6], but there is still no definite theory, so it is still of great significance to further study other factors involved in the occurrence and development of GC. Although *H. pylori* has been recognized as the most important carcinogen of GC and the initial factor of GC progression, eradication of *H. pylori* cannot completely prevent the occurrence of GC under the condition of gastric mucosal atrophy [7]. Moreover, there have been literature reports of advanced GC where *H. pylori* was in low abundance or absent [8]. In addition to *H. pylori*, there are a lot of other microorganisms in the stomach, which together constitute the microecology of the stomach. At present, there have been some studies on gastric microecology in patients with GC abroad [6, 9], indicating that the gastric microecology community has changed significantly during the development of GC.

With the development of high-throughput sequencing technology and metagenomics, many studies have shown that gastrointestinal microecology is closely related to the occurrence and development of GC [10, 11]. For example, in the long-term follow-up after gastrectomy, the fecal flora of patients with GC has changed, and the study of GC model mice found that antibiotic treatment on mice without *H. pylori* infection but with digestive tract symbiotic flora also played a role in the prevention of cancer [12, 13], indicating that there may be some other digestive tract bacteria also involved in the pathogenesis of GC. By comparing the rates of GC in germ-free mice, *H. pylori* mice and *H. pylori* bacteria-bearing mice, it was proved that digestive tract bacteria can also promote the carcinogenesis of *H. pylori*.

Therefore, the goal of further research should be to find bacteria closely related to GC except for *H. pylori*, so as to further study the pathogenesis of GC. A large number of microorganisms have been colonized in the intestines of healthy people. The intestinal microbial genome is considered as the second largest genome in the human body, and its relationship with diseases has always been a research hotspot. In recent years, studies have shown that intestinal microecology is closely related to many diseases, including rectal cancer, pancreatic cancer, type 2 diabetes, depression, Parkinson's disease, and cardiovascular disease [14–16]. On the other hand, some intestinal microorganisms play many beneficial roles, including immune system development, reaction or tolerance, participation in carbohydrate digestion, and toxic substance degradation. The beneficial bacteria that can help the body resist infection can competitively exclude endogenous and exogenous pathogens [17–19]. Moreover, the intestinal microbial community can effectively stimulate immune cells, which helps to start the human immune defense mechanism. Microorganisms and hosts constitute a completely functional ecosystem. Microorganisms provide immune stimulation, nutrition, and energy support for the host, and the host provides a colonization environment for microorganisms [20]. Some studies have found that the decrease of gastric acid secretion caused by atrophy of the gastric mucosa is conducive to the growth of gastric bacteria, thus promoting the synthesis of carcinogen *N*-nitrosamine. Several common intestinal microflorae infected with transgenic ins-gas sterile mice overexpressing human gastrin can promote the growth of gastric tumors in mice [21]. Another study found that this common intestinal microflora also had a high abundance in the stomach of patients with gastric precancerous lesions [22]. The researchers hypothesized these “intestinal flora” may be a high-risk factor for GC [23]. Therefore, it is very important to identify the key intestinal microflora associated with gastric cancer and maintain the stability of the core structure of intestinal microecology in patients with GC.

As an upper and lower connected lumen, there is a certain relationship between the flora of various digestive areas. Most of the bacteria colonized in the stomach can also be found in the oral cavity [24]. Because the intestinal tract is in the downstream channel of the gastric cavity, the occurrence of GC may have a significant impact on the intestinal flora of patients. However, there are few studies on the relationship between GC and the intestinal microbial community and the structural characteristics of intestinal flora in healthy and GC patients. The key gut microbiota specifically associated with gastric cancer remains unclear. Based on this, in order to study the relationship between gastrointestinal flora and GC as a whole, fecal samples of patients with GC were collected, and 16S rRNA sequencing technology was used to analyze the intestinal microecological structure and diversity characteristics of healthy people and GC patients, to find out the specific bacteria related to the intestinal microecology of GC patients, and further clarify the structural composition of intestinal microecology of GC patients. This method can be used as a new important way to prevent and treat surgical-related infections and provide a noninvasive and convenient means of diagnosis and monitoring for future disease research.

## 2. Materials and Methods

### 2.1. Sample Collection

From 32 to 76 years old, patients with gastric cancer confirmed by gastroscopy and histopathology for the first time in cancer surgery and without radiotherapy, chemotherapy, surgery, and other treatments were selected as the research objects, and healthy people who received routine gastroscopy in the hospital physical examination center were selected as the control group. The inclusion and exclusion criteria for patients with gastric cancer were that gastric cancer was first found under gastroscope and confirmed by histopathology after gastroscope and histopathology examination. The criteria for healthy people were that the gastric mucosa be normal under gastroscope and histopathology examination, without gastritis, peptic ulcer, and tumor focus. See Supplement Table S1 for specific patient information.

The experiment was divided into two groups: 30 fecal samples (*A*1) from gastric cancer patients and 30 fecal samples (*B*1) from healthy people were collected. Fresh feces were provided for patients with gastric cancer after diagnosis, and stool samples were provided for healthy people during physical examination. Fresh fecal samples were collected and loaded into sterile containers, then quickly put into the ice box and transferred to the laboratory for subpackaging. 200 mg fecal samples were accurately weighed, put into 2 ml sterile centrifuge tubes, divided into 5 parts, and stored in a −80°C refrigerator for testing.

### 2.2. Determination of Physiological and Biochemical Indexes and Pathological Observation

The physiological and biochemical indexes of the patient's blood samples were determined by a hematology analyzer, and the gastric tissue was observed by the H&E section and analyzed for the pathological structure.

### 2.3. DNA Extraction, PCR Amplification, and Sequencing

Total genome DNA from samples was extracted using the CTAB method. Primers were designed as follows: 515 forward (5′-GTGCCAGCMGCCGCGG-3′) and 806 reverse (5′-GGACTACHVGGGTWTCTAAT-3′) were used to amplify the V4 region of the 16S rDNA gene. PCR reactions were carried out with 15 *μ*L of Phusion® High-Fidelity PCR Master Mix (New England Biolabs); 2 *μ*M of forward and reverse primers; and about 10 ng template DNA. Thermal cycling consisted of initial denaturation at 98°C for 1 min, followed by 30 cycles of denaturation at 98°C for 10 s, annealing at 50°C for 30 s, and elongation at 72°C for 30 s. Finally, 72°C for 5 min. Sequencing libraries were generated using the TruSeq® DNA PCR-Free Sample Preparation Kit (Illumina, USA) following the manufacturer's recommendations and index codes were added. The library was sequenced on an Illumina NovaSeq platform.

### 2.4. Bioinformatics and Statistical Analyses

Sequence analysis was performed by Uparse software (Uparse v7.0.1001). Sequences with ≥97% similarity were assigned to the same OTUs. In order to study the phylogenetic relationship of different OTUs, and the difference between the dominant species in different groups, multiple sequence alignments were conducted using the MUSCLE software (Version 3.8.31). Alpha diversity is applied in analyzing the complexity of species diversity for a sample through 5 indices, including Chao1, Shannon, and Good-coverage. All these indices in our samples were calculated with QIIME (Version 1.7.0) and displayed with *R* software (Version 2.15.3). Beta diversity on both weighted and unweighted UniFrac was calculated by QIIME software (Version 1.9.1). The principal coordinate analysis (PCoA) was displayed by the WGCNA package, stat packages, and ggplot2 package in *R* software (Version 2.15.3). Unweighted pair-group method with arithmetic means (UPGMA) clustering was performed by using average linkage and was conducted by QIIME software (Version 1.9.1). The metastatic method was used to test the species abundance data among groups to get the *p* value, and then the *Q* value was obtained by correcting the *p* value. The species with significant differences were selected according to the Q value, and the distribution box diagram of the species diversity among groups was drawn. Linear discriminant analysis (LDA) effect size (LEfSe) was shown to identify taxa with significant differential abundances between samples. The factorial Kruskal–Wallis sum-rank test (*α* = 0.05) was used in LEfSe analysis, which was followed by LDA to estimate the effect size of each differentially abundant feature (logarithmic LDA score >2.0).

## 3. Results

### 3.1. The Overall Composition of Intestinal Flora in Patients with GC

**Figure 1:**
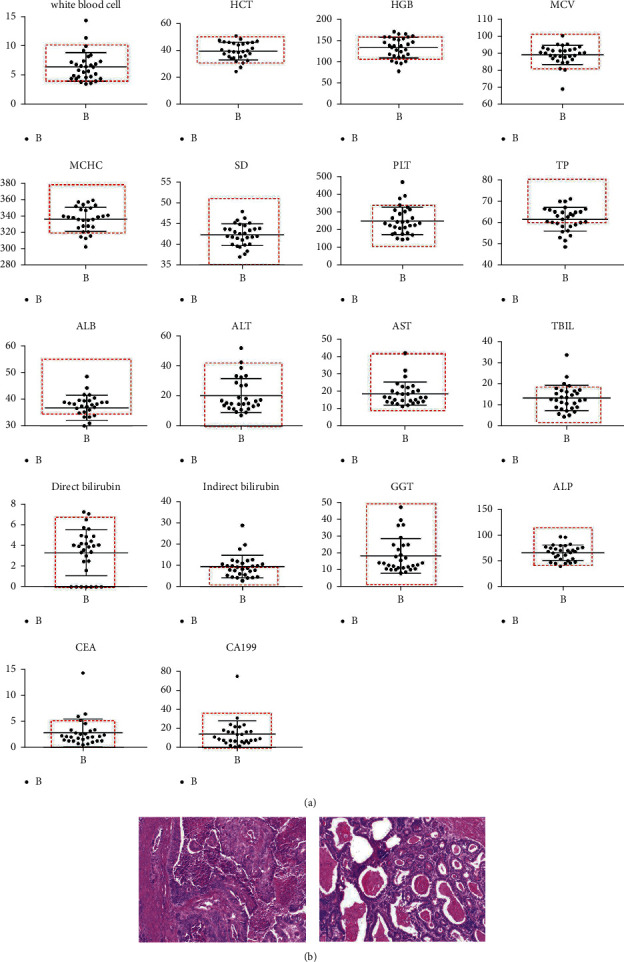
Blood routine analysis and pathological observation. (a) The content and distribution of WBC, HTC, HGB, MCV, SD, PLT, TP, ALB, ALT, AST, TBIL, direct bilirubin, indirect bilirubin, GGT, ALP, CEA, and CA199 in patients' blood were measured by hematology analyzer. (b) *H* & *E* section of the stomach in healthy people and patients with gastric cancer 200x.

We found that the content and distribution of WBC, HTC, HGB, MCV, SD, PLT, ALB, alt, AST, TBIL, direct bilirubin, GGT, ALP, CEA, and CA199 in group B were within the normal range (Figure 1(a)), indicating that there was no significant change in the blood routine of patients with gastric cancer, and the content and distribution of total protein TP were generally lower than the normal range, In addition, we also measured the contents of tumor markers CEA and CA199. The results showed that CEA <5 ug/L and CA199 <37 ku/L in these 30 patients. CA199, as an oligosaccharide tumor-associated antigen, is a new tumor marker and glycolipid on the cell membrane. It is the most sensitive marker for pancreatic cancer reported so far, but the analysis of CEA and CA199 in patients with gastric cancer showed that there was no significant correlation between the two tumor markers and the occurrence of gastric cancer, which indicated that they could not be used as one of the reference indicators for gastric cancer.

We observed the pathological changes of the *H* & *E* section of gastric tissue samples (Figure 1(b)) and found that compared with healthy people, there were poorly differentiated tumor tissues in the gastric antrum of patients with gastric cancer. The tumor cells showed nest-like distribution, with a large nucleus, deep staining, and obvious nucleolus. The cancer tissue invaded outside the serosa. There was poorly differentiated adenocarcinoma in some parts of the stomach, mucinous adenocarcinoma in some areas, and the tumor volume was 8.5 cm × 7 cm × 1.5 cm, the cancer tissue invaded the whole layer of the gastric wall, nerve invasion, and intravascular tumor thrombus were visible.

### 3.2. The Overall Composition of Intestinal Flora in Patients with GC

**Figure 2:**
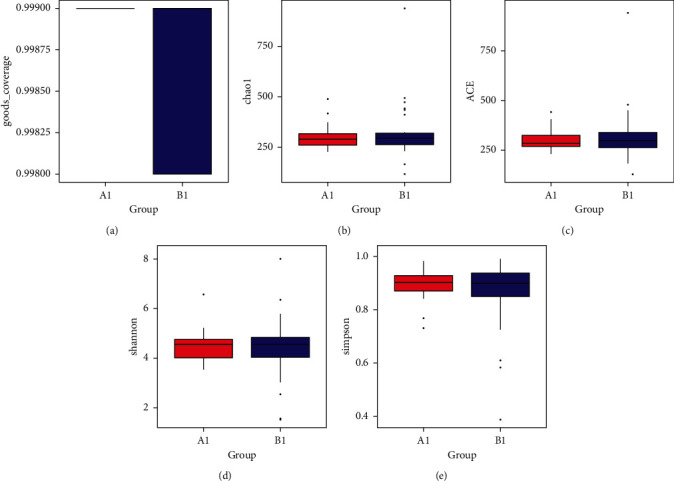
The overall microbial composition and diversity of GC patients and healthy people. (a)–(e) The good's coverage, Chao1 index, ACE, Shannon, and Simpson index for the two groups, and the significant differences between groups were calculated by Wilcoxon tests.

Good's coverage for the two groups was greater than 99.8%, indicating a great sequencing depth for the analysis of microbiota (Figure 2(a)). For alpha-diversity analysis, the Chao 1 and ACE estimators, as well as the Shannon and Simpson indexes were used to assessing community richness and diversity, respectively. It can be seen from the Chao 1 index and ACE index in Figures 2(b) and 2(c) that there was no significant difference between group *A*1 and group *B*1, indicating that the intestinal flora of patients with GC was like that of healthy people in terms of flora abundance, and there was no significant difference, that is, GC did not significantly change the abundance of intestinal flora. We also analyzed the diversity of intestinal flora in patients with GC by the Shannon and Simpson index and found that there was no significant difference between group *A*1 and group *B*1 (Figures 2(d) and 2(e)), indicating that the diversity of intestinal flora in patients with GC was similar to that in healthy people; that is, GC will not significantly change the diversity of intestinal flora. In general, the abundance and diversity of intestinal flora of patients with GC were not significantly different from those of healthy people, and GC cannot significantly change the abundance and diversity of intestinal flora.

**Figure 3:**
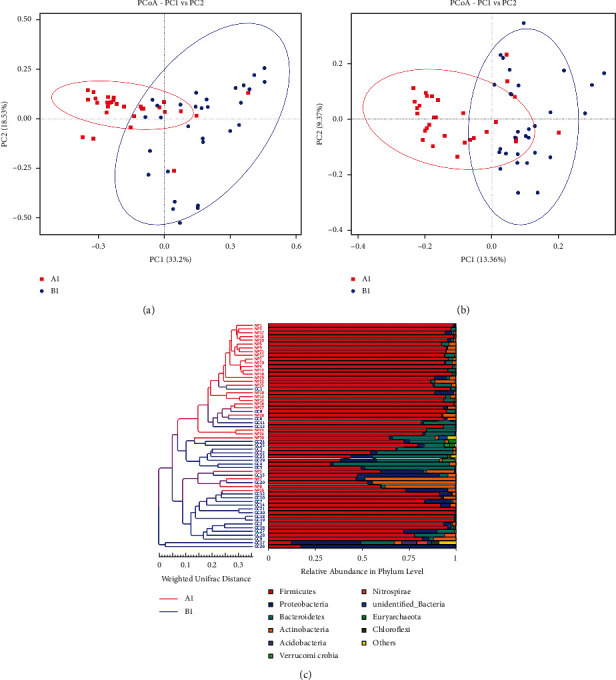
Similarities and differences in the overall distribution of intestinal flora between GC patients and healthy people. (a)-(b) PCoA plots based on weighted and unweighted UniFrac distances are colored by different groups. The significant differences between groups were calculated by analysis of similar (ANOSIM) tests; (c) UPGMA analysis based on unweighted UniFrac distances represented the similarity of intestinal flora distribution among different strains.

To measure the extent of the similarity between microbial communities in the two groups, principal coordinates analysis (PCoA) plots of weighted and unweighted distance were generated (Figures 3(a) and 3(b)). From the PCoA analysis, we found that there was a significant difference in microbial distribution between group *A*1 and group *B*1, among these algorithms, the PC1 distribution with the largest contribution rate revealed changes of 33.2% and 13.36%, and the PC2 distribution revealed changes of 18.53% and 9.37%. Then we used the unweighted pair-group method with arithmetic mean (UPGMA) to cluster the samples to determine the similarity of species composition among the samples. The closer the samples are, the more similar the species composition of the two samples is. It can be seen from Figure 3(c) that the composition of the intestinal flora of healthy people tends to gather together, while that of GC patients tends to be together. This result not only indicated the accuracy of sampling and testing; that is, the difference of samples within the group was small and the repeatability was high but also showed that the specific composition of intestinal flora between GC patients and healthy people was significantly different.

### 3.3. The Composition of the Intestinal Flora at the Phylum and Genus Level in GC Patients and Healthy People

**Figure 4:**
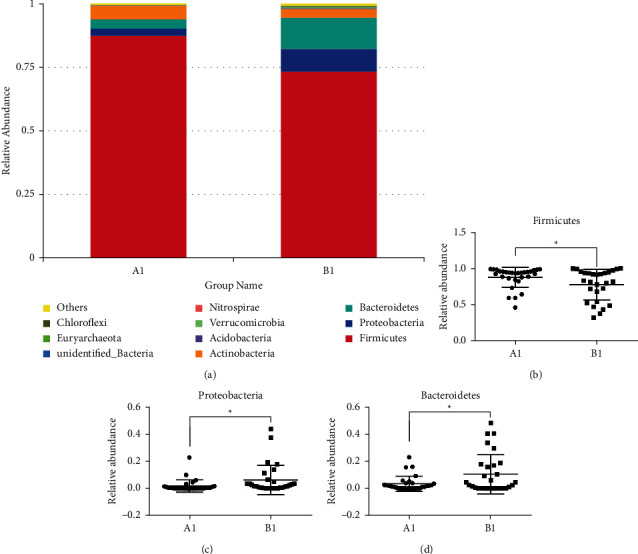
The specific composition of intestinal flora in GC patients and healthy people at the phyla level. (a) The relative contribution of the top 10 phyla in each group; (b)–(d) represented the relative abundance of firmicutes, proteobacteria, and bacteroidetes in the two groups, respectively, and data were analyzed by ANOVA (^*∗*^*p* < 0.05; ^*∗∗*^*p* < 0.01).

In order to explore the specific distribution and composition of the intestinal flora in GC patients and healthy people, we further analyzed the composition of the flora at the phylum level and the genus level. It can be seen from Figure 4(a) that the intestinal flora at the phylum level in group *A*1 was mainly composed of 87.7% *Firmicutes*, 2.7% *Proteobacteria*, 3.5% *Bacteroidetes,* and 5.4% *Actinobacteria*, and the intestinal flora at the phylum level in group *B*1 was mainly composed of 73.4% *Firmicutes*, 9.0% *Proteobacteria*, 12.3% *Bacteroidetes,* and 3.5% *Actinobacteria*. Through one-way analysis of variance we found that the relative abundance of *Firmicutes* in the *A*1 group was significantly higher than that of the *B*1 group (*p* < 0.05, Figure 4(b)), and the relative abundance of *Proteobacteria* and *Bacteroidetes* in the *B*1 group were significantly higher than those in group *A*1 (*p* < 0.05, Figures 4(c) and 4(d)), that is, GC would significantly reduce the abundance of *Firmicutes* in the intestine, and significantly increase the abundance of *Proteobacteria* and *Bacteroidetes.*

**Figure 5:**
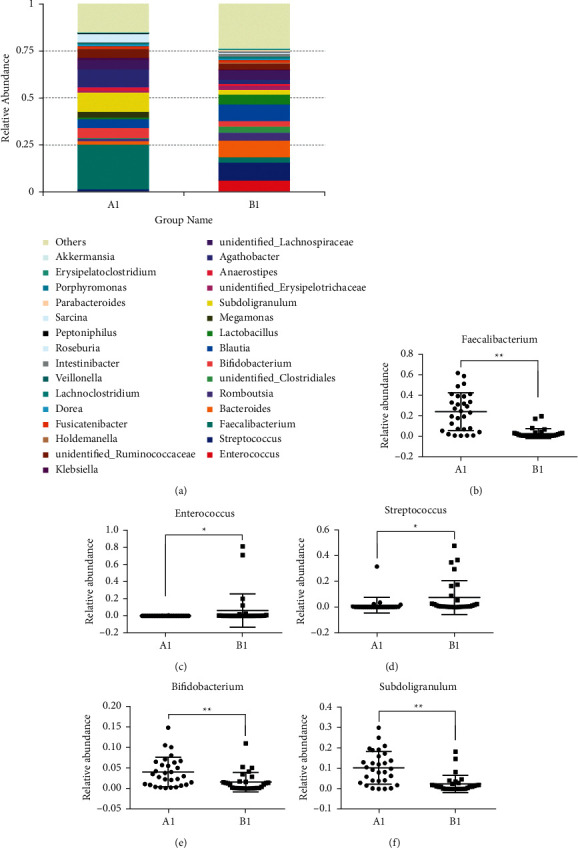
The specific composition of intestinal flora in GC patients and healthy people at the genera level. (a) The relative contribution of the top 30 genera in each group; (b)–(d) represented the relative abundance of *Faecalibacterium*, *Enterococcus*, *Bacteroides*, *Bifidobacterium*, and subdoligranulum in the two groups, respectively, and data were analyzed by ANOVA (^*∗*^*p* < 0.05; ^*∗∗*^*p* < 0.01).

Through the analysis of the genus level of the intestinal flora (Figure 5(a)), we found that the *A*1 group was mainly composed of *Faecalibacterium*, *Bifidobacterium*, *Blautia*, *Megamonas*, *Subdoligranulum,* and *Agathobacter*, and the *B*1 group was mainly composed of *Enterococcus*, *Streptococcus*, *Bacteroides*, *Blautia,* and *Lactobacillus*. Through one-way analysis of variance, it was found that the relative abundance of *Faecalibacterium* in the *A*1 group was significantly higher than that of the *B*1 group (*p* < 0.01, Figure 5(b)), and the relative abundances of *Enterococcus* and *Streptococcus* in the *B*1 group were significantly higher than those in the *A*1 group (*p* < 0.05, Figures 5(c) and 5(d)), while in the *A*1 group the relative abundances of *Bifidobacterium* and *Subdoligranulum* were significantly higher than those of group *B*1 (*p* < 0.01, Figures 5(e) and 5(f)). The above results indicated that the composition of intestinal microflora in patients with GC was significantly different from that in healthy people. GC can significantly reduce the abundance of *Faecalibacterium*, *Bifidobacterium,* and *Subdigranulum*, and significantly increase the abundance of *Enterococcus*, *Streptococcus,* and *Bacteroides*.

### 3.4. The Differences in the Dominant Members of the Microbiota

**Figure 6:**
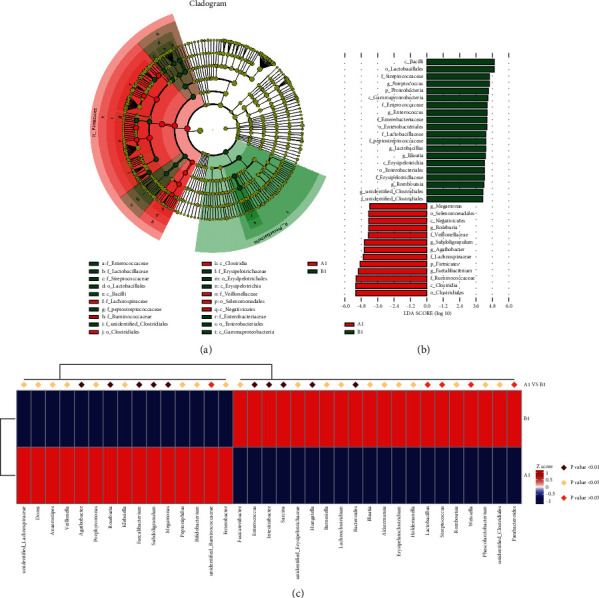
Major differential microbial species in GC patients and healthy people. (a) Taxonomic cladogram obtained from LEfSe at the two groups. Biomarker taxa are highlighted with colored circles and shaded areas. Each circle's diameter reflects the abundance of those taxa in the community; (b) taxonomic cladogram obtained from the linear discriminant analysis (LDA) at the two groups, the cutoff value of ≥2.0 used.

In order to verify and further determine the more different significant microorganisms in each group, we also conducted LEfSe analysis. LEfSe was used to identify the specific phylotypes responding to these groups. As shown in Figures 6(a) and 6(b), through the LEfSe analysis of the *A*1 group and *B*1 group, we found that compared with the *A*1 group, the significantly different microorganisms in the *B*1 group were *Lactobacillales*, *Streptococcaceae*, *Streptococcus*, *Proteobacteria*, *Enterococcaceae*, *Enterobacteriales*, *Enterococcus*, *Peptostreptococcaceae*, *Blautia,* and *Roseburia*; the difference microorganisms enriched in the *A*1 group were *Megamonas*, *Selenomonadales*, *Roseburia*, *Subdoligranulum*, *Agathobacter*, *Lachnospiraceae*, *Faecalibacterium*, *Ruminococcaceae,* and *Clostridiales*. We performed metastat analysis on the results of the intestinal flora of the two samples (Figure 6(c)), and found that compared with the *B*1 group, the enriched microorganisms with significant differences in *A*1 were *Agathobacter*, *Roseburia*, *Faecalibacterium*, *Subdoligranulum,* and *Megamonas* (*p* < 0.01); the enriched microorganisms with significant differences in B1 group included *Enterococcus*, *Intestinibacter, Sarcina*, *Hungatella,* and *Bacteroides* (*p* < 0.01).

### 3.5. Functional Gene Prediction

**Figure 7:**
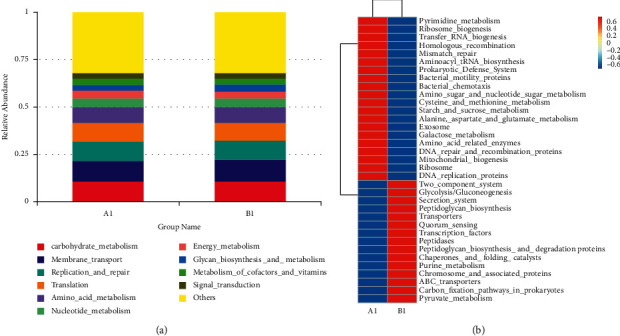
Functional gene prediction. (a)-(b) Prediction heatmap of functional genes based on Tax4Fun under L3 pathways at the two groups.

The intestinal flora plays a certain role in the intestine and is closely related to the function of the body. Since we found that there were significant differences in intestinal flora between GC patients and healthy people through 16S rRNA sequencing technology, we further analyzed and predicted the functions between different groups. Based on Tax4Fun (Figures 7(a) and 7(b)), we found that the functional genes of the intestinal flora in the *A*1 and *B*1 groups were mainly expressed in the following pathways: Carbohydrate_metabolism, Membrane_transport, Replication_and_repair, Translation, Amino_acid_metabolism, Nucleotide_metabolism, Energy_metabolism, Glycan_biosynthesis_and_meyabolism, Metabolism_of_cofactors_and_vitamins, and Signal_transduction. Among them, Carbohydrate_ metabolism, Membrane_ transport, Replication_ and_ Repair, Translation, and Amino_ acid_ Metabolism were the most expressed; we further analyzed the specific differential metabolic pathways between the GC group and the healthy group and found that the differential function expression of the intestinal flora of healthy people were mainly rich in pyrimidine_metabolism, ribosome_biogenesis, transfer_RNA_biogenesis, homologous_recombination, mismatch_repair, aminoacyl_tRNA_biosynthesis, prokaryotic_defense_system, bacterial_motility_proteins, amino_sugar_and_nucleotide_sugar_metabolism, cysteine_and_methionine_metabolism, starch_and_sucrose_metabolism, alanine, _aspartate_and_glutamate_metabolism, exosome, galactose_metabolism, amino_acid_related_enzymes, DNA_repair_and_recombination_proteins, mitochondrial_biogenesis, bacterial_chemotaxis, ribosome, and DNA_replication_proteins. The differential function expression of the intestinal flora of GC patients were mainly rich in two_component_system, glycolysis/gluconeogenesis, secretion_system, peptidoglycan_biosynthesis, transporters, quorum_sensing, transcription_factors, purine_metabolism, ABC_transporters, peptidases, chaperones_and_folding_catalysts, chromosome_and_associated_proteins, carbon_fixation_pathways_in_prokaryotes, peptidoglycan_biosynthesis_and_degradation_proteins, and pyruvate_metabolism.

## 4. Discussion

At present, most of the studies on gastric microbial communities use gastric mucosal samples obtained by surgical resection or biopsy to explore the differences and changes in gastric microbial communities [25, 26]. However, there are few reports on intestinal microecology in patients with GC. As the intestinal tract is in the downstream channel of the gastric cavity, the occurrence of GC may have a significant impact on the intestinal flora of patients. Therefore, in this study, we used more convenient and noninvasive fecal samples to compare the composition and diversity of the intestinal microbial community between GC patients and healthy people by using 16S rRNA technology to study the structure and diversity of intestinal flora in patients with GC. We found that there were significant differences in the composition of intestinal flora between GC patients and healthy people. *Firmicutes*, *Proteobacteria,* and *Bacteroidetes* were the main phyla in the intestinal microflora of GC patients. This result was consistent with studies on the microbial community in the stomach of patients with GC in the literature [23, 27]. At the same time, we found that the relative abundance of *Firmicutes* in GC patients was significantly lower than that in healthy people, while the relative abundance of *Proteobacteria* and *Bacteroidetes* were significantly higher than that of healthy people.

In bioinformatics, the Chao 1 and ACE indexes can reflect the abundance of bacteria. The Shannon index is a more comprehensive parameter to describe the abundance and uniformity of a microbial community. This study found that the abundance and diversity of intestinal microflora in patients with GC were similar to that of healthy people, and there was no significant difference. This also indicated that GC may have little impact on the abundance and diversity of intestinal flora in the lower reaches of the digestive tract. In some previous studies, it was considered that the intestinal microflora of GC patients had higher abundance and a lower Shannon index. Many factors, such as digestive tract oxygen concentration, intraluminal pH value, and drug use, may change the intestinal microecological diversity [28]. The decrease in the Shannon index represents the change in intestinal microecology diversity in patients with GC. Previous studies revealed that the decrease in the microecological diversity index may be a biomarker of gastrointestinal inflammation and cancer [29]. In general, this study found that the diversity and abundance of intestinal flora in patients with GC may not be a relevant indicator for accurate detection of early GC.

In addition, gastric acid can be considered as the most important factor for bacterial colonization and growth in the digestive tract [30]. Beasley et al. [31] reported that gastric acid can rescreen the microecology of gastric contents before they enter the intestinal tract. During the formation of GC, gastric acid secretion gradually decreased [32], which may lead to the formation of a relatively mild acid environment in the intestinal tract of patients with GC, and the formation of a unique intestinal microecology. In this study, we found that the relative abundance of *Faecalibacterium*, *Bifidobacterium,* and *Suboligranulum* in the intestinal tract of patients with GC were significantly lower than that in healthy subjects, while the relative abundance of *Enterococcus*, *Streptococcus,* and *Bacteroides* were increased. *Faecalibacterium* is a genus of clostridial, which is one of the most important symbionts in human intestinal microflora. Gopalakrishnan et al. [33] found that patients with a high abundance of *Faecalibacterium* had higher levels of circulating CD4+ and CD8+ cells as well as cytokines with synergistic antiPD-1 drugs. From the perspective of antigen processing and presentation in the tumor microenvironment, it was found that the density of immune cells and antigen processing markers presented by patients with *Faecalibacterium* in the intestinal microbiota group was higher than that in patients with rich *Bacteroidetes*. Watanabe et al. [5] found that *Faecalibacterium* was absent in gastric biopsy samples of GC patients with positive *Helicobacter pylori* infection. They thought *H. pylori*-positive significantly affected the composition and distribution of microorganisms, indicating that GC had certain inhibition and influence on the colonization of *Faecalibacterium.*

At present, it is believed that the change of intestinal flora is closely related to GC. The change in intestinal bacterial composition will destroy the physiological interaction of microbiota and be related to the intestinal immune system, leading to chronic inflammation and cancer. The relationship between the gut microbiota and GC is thought to be that the gut microbiota can continuously activate the host's immune system. The final result of the imbalance of the interaction between host epithelial cells and microorganisms is chronic inflammation. At the same time, the intestinal flora promotes an antitumor immune response through a variety of mechanisms [2]. GC leads to the imbalance of normal intestinal flora, and the disturbance of intestinal microecology can lead to the invasion, proliferation, and growth of pathogenic bacteria and destroy the homeostasis of the immune system and mucosal barrier. The subsequent inflammatory process leads to the increase of intestinal permeability, which makes intestinal microbes drive a persistent inflammatory state and activate TLR, NLR, and YAP1 signals, causing endotoxin and other harmful substances to enter the blood through the intestine, causing an inflammatory reaction and cancer [34, 35]. This theory is consistent with our findings in this study. We found that *Enterococcus* was significantly increased in GC patients, while the relative abundance of *Bifidobacterium* was decreased. *Enterococcus* is an important pathogen of infection in patients. It can not only cause urinary tract infections, skin infections, and soft tissue infections but also cause life-threatening abdominal infection, sepsis, pericarditis, and meningitis. *Enterococcus* can produce many factors related to pathological changes in the host [36]. Polymorphonuclear leukocyte chemokines produced by *Enterococcus faecalis* can mediate, or at least partially mediate, the inflammatory response usually associated with enterococcal infection. *Enterococcus* can also produce a plasmid-encoded hemolysin that can increase the severity of infection. In addition, *Enterococcus* can induce platelet aggregation and cytokine-dependent fibrin production, which may be related to the pathogenesis of enterococcal endocarditis [37]. The increase in the relative abundance of *Enterococcus* may be related to intestinal preparation, drug use, and other factors in patients with GC before the operation, and may also be related to the mechanism of GC. That is, GC can lead to the increase of *Enterococcus*. The relationship between *Enterococcus* and GC can be further studied. Strickertsson et al. [38] explored the relationship between *Enterococcus faecalis* and human GC cells and found that the infection of *Enterococcus faecalis* led to the instability of mitochondrial DNA in GC cells, and the transcription of genes encoding inflammatory response protein was up-regulated, while DNA damage repair and cell cycle control genes were downregulated. That is, *Enterococcus faecalis* infection can increase the inflammatory response of gastric cells and destroy the mitochondrial genome. Finally, the bacteria-induced NF-*κ*B inflammation, DNA damage, and cell cycle control gene expression all showed up. *Bifidobacterium*, as an important intestinal beneficial microorganism, has many important physiological functions on human health, such as biological barrier, nutrition, antitumor, immune enhancement, improvement of gastrointestinal function, antiaging and other important physiological functions, and has a very close relationship with human health and diseases [39]. *Bifidobacterium* and other beneficial bacteria can inhibit the growth of harmful bacteria in the human intestine, resist the infection of pathogenic bacteria, synthesize vitamins needed by the human body, promote the absorption of minerals, produce organic acids such as acetic acid, propionic acid, butyric acid, and lactic acid, stimulate intestinal peristalsis, promote defecation, prevent constipation, inhibit intestinal corruption, purify the intestinal environment, and decompose carcinogens. Researchers have reported that PD-L1 or PD-L1^P146R^ is associated with tumorigenesis and poor prognosis of gastric cancer, while *Bifidobacterium* could promote antitumor immunity and facilitate anti-PD-L1 efficacy in gastric cancer [40–42]. Therefore, *Bifidobacterium* plays an important role in stimulating the human immune system and improving disease resistance. In this study, we found that the abundance of *Bifidobacterium* in GC patients was significantly decreased, which indirectly indicated that the intestinal immune function of GC patients was reduced and decreased. Chen et al. [43] found that the *Bifidobacterium* extracellular polysaccharide significantly inhibited the growth of human GC cell line BGC-823 and had a certain effect on the telomerase rate limiting factor, human telomerase reverse transcriptase activity. However, the concentration of *bifidobacterium* in our study needs further investigation since the number of samples for gut microbiota measurements was limited. Hou et al. [44] successfully measured the concentration of *bifidobacterium* in feces samples using a highly sensitive quartz crystal microbalance immunosensor, which brings new implications for our future study.

## 5. Conclusion

In general, we found that these six kinds of intestinal bacteria *Faecalibacterium*, *Bifidobacterium,* Subdoligranulum*, Enterococcus*, *Streptococcus,* and *Bacteroides* were related to the occurrence and risk of GC, which had a good diagnostic value for distinguishing normal people from patients and can be used as a direction and idea for the prevention and diagnosis of gastric cancer.

## Data Availability

The data sets used and/or analyzed during the present study are available from the corresponding author on reasonable request.

## Supplementary Materials

Supplementary Materials Table S1. characteristics and quality scores of the studies.
