# Supplementary material for: Explore and Analyze the Composition and Characteristics of Intestinal Microbiota between Gastric Cancer Patients and Healthy People
Source: Evid Based Complement Alternat Med. 2022 Sep 8;2022:5834293. doi: 10.1155/2022/5834293 (PMC9477631; doi:10.1155/2022/5834293)
Supplement: Supplementary Materials — Table S1. characteristics and quality scores of the studies. [file 5834293.f1.docx]

**Table S1. characteristics and quality score of the studies.**

| Serial Number | Sex | Age | Diagnosis | Family History | Drinking | Smoking |
| --- | --- | --- | --- | --- | --- | --- |
| 1 | Female | 74 | Carcinoma of gastric antrum (T2N2M0) | none | none | none |
| 2 | male | 47 | Carcinoma of gastric antrum(T2N1M0) | none | 30 years of drinking | 30 years of smoking |
| 3 | male | 74 | Carcinoma of gastric antrum(Borrmann Ⅳ,T4) | none | none | none |
| 4 | male | 55 | Carcinoma of gastric antrum(T4bN3M0) | His mather died of esophageal cancer | Light drinking | 30 years of smoking |
| 5 | male | 58 | gastric cardiac adenocarcinoma(T4N3M0) | none | none | none |
| 6 | male | 63 | Antral carcinoma of stomach(T4N2M0) | none | none | 30 years of smoking |
| 7 | male | 61 | Carcinoma of gastric antrum(T3N1M0) | none | 15 years of drinking | 20 years of smoking |
| 8 | male | 52 | Carcinoma of gastric antrum (T4N2M0) | none | none | none |
| 9 | Female | 57 | Carcinoma of gastric antrum(T4N3M0) | none | none | none |
| 10 | male | 69 | Carcinoma of gastric horn(T4N1M1) | none | none | 50 years of smoking |
| 11 | male | 74 | Carcinoma of gastric antrum(T1N1M0) | none | none | none |
| 12 | male | 71 | Poorly differentiated adenocarcinoma of gastric antrum(T2N1M0) | none | Light drinking | 40 years of smoking |
| 13 | male | 75 | Carcinoma of gastric horn(T1N2M0) | none | none | 50 years of smoking |
| 14 | male | 48 | Carcinoma of gastric antrum(T4N3M0) | none | none | none |
| 15 | male | 68 | Carcinoma of gastric horn(T4N3M0) | none | 20 years of drinking | 40 years of smoking |
| 16 | male | 63 | Carcinoma of gastric antrum(T4N3M0) | none | none | none |
| 17 | Female | 57 | Carcinoma of gastric antrum(T4N2M0) | none | none | none |
| 18 | male | 43 | Poorly differentiated adenocarcinoma of gastric antrum | none | none | 15 years of smoking |
| 19 | Female | 32 | Carcinoma of gastric horn(T2M0N0) | none | none | none |
| 20 | male | 76 | Carcinoma of gastric antrum(T4N3M0) | none | none | none |
| 21 | Female | 55 | Poorly differentiated adenocarcinoma of gastric antrum (T1bN0M0)； | His father died of esophageal cancer | none | none |
| 22 | male | 66 | Moderately differentiated adenocarcinoma of gastric antrum(T1N0M0) | His father died of stomach trouble | none | 10 years of smoking |
| 23 | Female | 72 | Carcinoma of gastric antrum | none | none | none |
| 24 | Female | 62 | Carcinoma of gastric antrum(T4aN3M0) | none | none | none |
| 25 | Female | 45 | Carcinoma of gastric horn(T4aNOMO) | none | none | none |
| 26 | male | 66 | Carcinoma of gastric antrum(T4N3M0) | none | none | none |
| 27 | male | 71 | gastric cardiac adenocarcinoma | none | none | none |
| 28 | male | 58 | gastric cardiac adenocarcinoma(T4N3M0 ) | none | none | none |
| 29 | male | 73 | Well differentiated adenocarcinoma of gastric antrum(T1N0M0) | none | none | none |
| 30 | Female | 71 | Carcinoma of gastric horn(T2N1MO) | none | none | none |
